# Identification and characterization of repetitive DNA in the genus *Didelphis* Linnaeus, 1758 (Didelphimorphia, Didelphidae) and the use of satellite DNAs as phylogenetic markers

**DOI:** 10.1590/1678-4685-GMB-2020-0384

**Published:** 2021-04-16

**Authors:** Cayo Augusto Rocha Dias, Gustavo C. S. Kuhn, Marta Svartman, José Eustáquio dos Santos, Fabrício Rodrigues Santos, Christian Miguel Pinto, Fernando Araújo Perini

**Affiliations:** 1 Universidade Federal de Minas Gerais, Instituto de Ciências Biológicas, Laboratório de Evolução de Mamíferos, Belo Horizonte, MG, Brazil.; 2 Universidade Federal de Minas Gerais, Instituto de Ciências Biológicas, Laboratório de Citogenômica Evolutiva, Belo Horizonte, MG, Brazil.; 3 Universidade Federal de Minas Gerais, Instituto de Ciências Biológicas, Laboratório de Biodiversidade e Evolução Molecular, Belo Horizonte, MG, Brazil.; 4 Escuela Politécnica Nacional, Facultad de Ciencias, Departamento de Biologia, Quito, Ecuador.

**Keywords:** RepeatExplorer, retrotransposons, satellite DNA, Didelphini

## Abstract

*Didelphis* species have been shown to exhibit very conservative karyotypes, which mainly differ in their constitutive heterochromatin, known to be mostly composed by repetitive DNAs. In this study, we used genome skimming data combined with computational pipelines to identify the most abundant repetitive DNA families of *Lutreolina crassicaudata* and all six *Didelphis* species. We found that transposable elements (TEs), particularly LINE-1, endogenous retroviruses, and SINEs, are the most abundant mobile elements in the studied species*.* Despite overall similar TE proportions, we report that species of the *D. albiventris* group consistently present a less diverse TE composition and smaller proportions of LINEs and LTRs in their genomes than other studied species. We also identified four new putative satDNAs (sat206, sat907, sat1430 and sat2324) in the genomes of *Didelphis* species*,* which show differences in abundance and nucleotide composition. Phylogenies based on satDNA sequences showed well supported relationships at the species (sat1430) and groups of species (sat206) level, recovering topologies congruent with previous studies. Our study is one of the first attempts to present a characterization of the most abundant families of repetitive DNAs of *Lutreolina* and *Didelphis* species providing insights into the repetitive DNA composition in the genome landscape of American marsupials.

## Introduction

The genus *Didelphis* Linnaeus, 1758 comprises six species of medium-sized American marsupials (Astúa, 2015a; Cerqueira and Tribe, 2008): *Didelphis virginiana* Kerr, 1792, inhabiting tropical, subtropical, and temperate zones of North America, and two groups of species found in tropical and subtropical zones of Neotropics (Astúa, 2015a; Cerqueira, 1985). The *D. albiventris* group (white-eared opossums) consists of three species found exclusively in South America: *D. albiventris* Lund, 1840, *D. imperfecta* Mondolfi and Pérez-Hernández, 1984, and *D. pernigra* J. A. Allen, 1900. The *D. marsupialis* group (black-eared opossums) is composed of two Neotropical species: *D. aurita* Wied-Newied, 1826 and *D. marsupialis* Linnaeus, 1758 (Cerqueira and Tribe, 2008). Despite showing skull morphometric differences among them when a restricted set of *Didelphis* species are studied (Cerqueira and Lemos, 2000; Lemos and Cerqueira, 2002; Ventura et al., 2002), overlapping skull shape and size are observed when all species are compared simultaneously (Astúa, 2015b). Although most molecular-based phylogenies also failed to provide significant support for the relationships among white-eared opossums (Amador and Giannini, 2016; Dias and Perini, 2018), a recent mitogenome-based phylogeny seem to provide support and resolution to the relationships among *Didelphis* species of this group (Dias *et al*., 2020).

*Didelphis* species are also cytogenetically conserved, presenting only a single diploid number, 2n = 22, a trait that is shared with other members of the Didelphini tribe (Yonenaga-Yassuda et al., 1982; Svartman and Vianna-Morgante, 1999). Cytogenetic studies employing banding techniques and chromosome painting have led to the suggestion that this cytogenetic conservation may extend to the whole chromosome composition, with species differences mainly residing in the heterochromatin (Yonenaga-Yassuda *et al*., 1982; Svartman and Vianna-Morgante, 1999, 2003).

Heterochromatin is mainly composed of repetitive DNAs, primarily tandemly repeated satellite DNAs (satDNAs) and transposable elements (TEs) (retrotransposons and transposons) (López-Flores and Garrido-Ramos, 2012; Saksouk et al., 2015). Repetitive DNAs are also major components of eukaryotic genomes, notably in marsupials where they may represent more than 50 % of the genomes (Mikkelsen et al., 2007; Renfree *et al*., 2011). Variation in the abundance and composition of repetitive DNAs are likely the cause of the heterochromatin variation found across *Didelphis* species reported in previous cytogenetic studies. However, there are no data on repetitive DNAs in these species that could support this prediction.

The advent of high-throughput next-generation sequencing (NGS) provided a fast and cost-effective manner to produce sequence data that can be used to identify the most frequent genome components in a single sequencing run (Ansorge, 2009; Novák et al., 2010). This NGS output serves then as input for similarity and graph-based *in silico* analyses that have been proven to be an efficient strategy for *de novo* identification and characterization of repetitive DNAs (Garrido-Ramos, 2017; Novák *et al*., 2010, 2013, 2017; Silva et al., 2019).

In this study, we used Illumina NGS technologies to perform a low pass shot-gun genome sequencing of Didelphini taxa of all *Didelphis* species and *Lutreolina crassicaudata* Desmarest 1804, combined with computational pipelines in order to identify the most abundant repetitive DNA families. With this approach we aimed to characterize the identified putative satDNA families and assess their utility in the phylogenetic inference of the genus *Didelphis* (De La Herrán et al., 2001; López-Flores et al., 2004; Shubina et al., 2015), comparing it to previous molecular-based studies (e.g. Dias and Perini, 2018; Dias *et al*., 2020).

## Material and Methods

### Samples, DNA extraction and genome sequencing

We obtained tissue samples of all species of *Didelphis* as well of *Lutreolina crassicaudata* (included as an outgroup taxon) from taxonomic collections of the following institutions: Escuela Politécnica Nacional (*D. pernigra*), Kwata NGO, French Guiana *(D. imperfecta*), Royal Ontario Museum (*D. virginiana*), Universidade do Estado do Mato Grosso (*D. marsupialis*), Universidade Federal de Minas Gerais (*D. albiventris, D. aurita* and *L. crassicaudata*). The genomic DNA of each species was extracted using the phenol-chloroform protocol (Sambrook and Russel, 2001) and employed on the preparation of DNA libraries using the Nextera DNA Flex Library Prep kit according to the manufacturer’s instructions (Illumina Inc., San Diego, CA). A paired-end (2 x 150 bp) sequencing run was performed on a NextSeq system at Instituto René Rachou - Fiocruz Minas (Table S1).

### Identification and characterization of repetitive DNAs

We used FastQC 0.11.9 (Andrew, 2010) to perform quality control check on raw Illumina reads, which were submitted to fastp (Chen et al., 2018) for trimming and quality filtering using default settings. A random sample of processed reads of each species was subjected to two pipelines developed for identification and characterization of repetitive DNAs in unassembled next-generation sequencing data (NGS): RepeatExplorer 2 (Novák et al., 2010, 2013) and TAREAN (Novák *et al*., 2017). Both pipelines apply similarity and graph-based clustering methods to create clusters of reads corresponding to different repetitive DNA families. Analyses were performed using the versions of RepeatExplorer 2 and TAREAN implemented on the Galaxy platform (Goecks et al., 2010) and applying the long and low “queue” option. As an additional step, in both pipelines, reads of each cluster are assembled into contigs that can be used in protein domain search or manual annotation.

In order to obtain a more accurate classification of the repetitive DNAs found in the studied species, we used LAST 1080 (Kielbasa et al., 2011) to search for similarities between these contigs and the collection of previously identified repetitive elements form *Monodelphis domestica*, the only didelphid species whose genome has been sequenced and annotated, deposited in the Repbase database (Bao et al., 2015). We also used LAST 1080 to perform pairwise comparisons between the consensus sequences of the putative satDNAs identified by RepeatExplorer 2 and TAREAN and each of the contigs produced by these pipelines for each species. LAST 1080 results were then used as input for a custom python script designed to create a table summarizing the results and multi-fasta files encompassing contigs arranged by species and by putative satDNA family. Contigs were selected based on two criteria: alignment size > 100 bp and identity > 70 %. Each multi-fasta file generated on the previous step was aligned with MAFFT 7 (Katoh and Standley, 2013) using the E-INS-i and L-INS-i algorithms for sequences containing or not long insertion/deletions, respectively.

Aligned DNA sequences were visualized and edited with Aliview 1.26 (Larsson, 2014). Nucleotide composition and intraspecific genetic Kimura-2-parameter distance were estimated for each satDNA with MEGA X (Kumar et al., 2018). Additionally, a contig of each species and each satDNA was submitted to the online version of CENSOR (Kohany et al., 2006) in order to identify possible relationships between satDNAs and transposable elements. We also built self-similarity dot plots and dot plots for each pair of satDNA consensuses with Gepard 1.40 (Krumsiek et al., 2007) to check for similarities within and among them.

To assess the use of the putative satDNA sequences as potential taxonomic markers, maximum likelihood phylogenetic trees were constructed for each satDNA separately. Phylogenetic analyses were performed using IQTREE 1.6 (Nguyen et al., 2015) based on substitution models defined using its built-in model finder (Kalyaanamoorthy et al., 2017).

## Results

In order to investigate the most frequent families of repetitive DNAs in the genomes of *Didelphis* and *L. crassicaudata,* we used graph-based clustering methods employed by RepeatExplorer 2 and TAREAN. The overall genome proportion of repDNAs detected by these pipelines ranged from 10.85 % in *D. marsupialis* to 13.63 % in *D. imperfecta* (Table 1).

**Table 1 -:** Repetitive DNA composition estimated by RepeatExplorer 2 and TAREAN pipelines from Illumina short reads for *Didelphis* species and *Lutreolina crassicaudata.*

Repeat Type	*D. aurita*	*D. marsupialis*	*D. albiventris*	*D. imperfecta*	*D. pernigra*	*D. virginiana*	*L. crassicaudata*
Satellite DNA	0.04	0.42	3.64	2.52	1.14	0.34	0.21
Class I TE							
LINE	10.2	10.04	9.14	9.56	9.53	10.09	11.76
LTR							
Retrovirus	2.01	0.39	0.36	1.55	1.61	2.22	0.31
Total	12.25	10.85	13.14	13.63	12.28	12.65	12.28

### Transposable elements

RepeatExplorer 2 results indicated transposable elements (TE), particularly long interspersed elements (LINEs) and long terminal repeats (LTRs) retrotransposons, as the most abundant repetitive DNAs in all species. Overall, the estimated genome proportion of TEs in *Didelphis* ranged from 9.50 % (*D. albiventris*) to 12.31 % (*D. aurita*), and 12.07 % in *L. crassicaudata* (Table 1).

**Figure 1 -:**
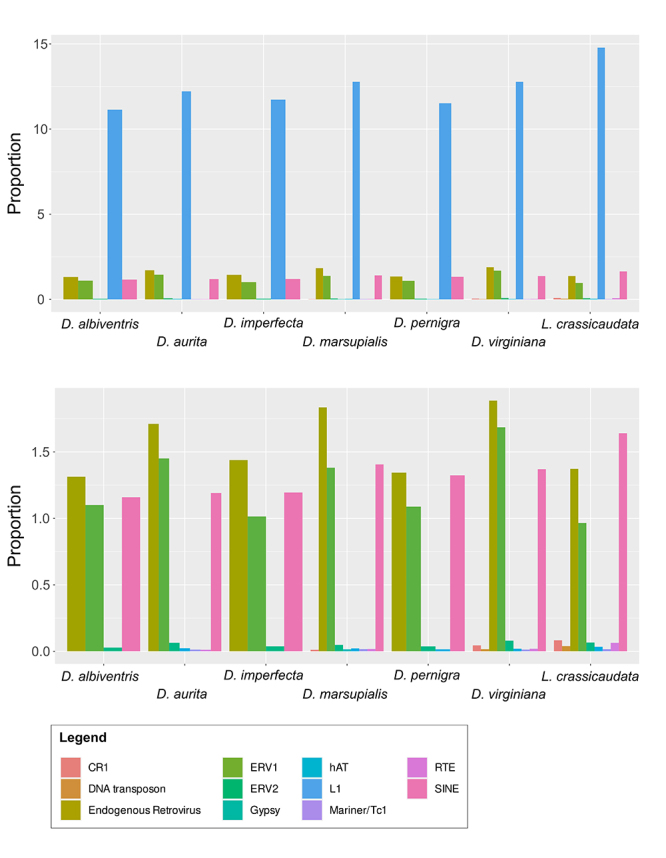
Bar-plot depicting the genome proportion of the transposable elements (TE) identified by the RepeatExplorer 2 pipeline (A) with LINE-L1 elements and (B) without LINE-L1 elements.

Similarity-based comparisons between contigs assembled as part of the RepeatExplorer 2 pipeline and the sequences from previously identified repetitive DNAs of *Monodelphis domestica* deposited in RepBase allowed the identification of 11 families of TEs with genome proportions above 0.01 %. Among them, LINE L1 was the most abundant in all studied species with genome proportions ranging from 11.14 % (*D. albiventris*) to 14.79 % (*L. crassicaudata*) (Figure 1). Families of endogenous retrovirus LTRs (*e.g.* ERV1 and ERV2) were also found in all species, being the second most abundant TE in *Didelphis* and the third most abundant in *L. crassicaudata*. Although LTR proportions exceed 3 % in *D. aurita, D. marsupialis* and *D. virginiana*, individual LTR families proportion did not reach 2 % (Figure 1 and Table S2). Both LINEs and LTRs are less abundant in the genomes of white-eared opossums. Short interspersed elements (SINEs) make up the third most abundant TE in all *Didelphis* species and the second one in *L. crassicaudata*. Less frequent types of TEs were only found in individual species, as was the case of the Gypsy LTR retrotransposon in *D. marsupialis* (0.02 %) (Figure 1 and Table S2). DNA transposon families such as Mariner/Tc1 and hAT were also found in most species. The former was not found in *D. albiventris* and *D. imperfecta* whereas the latter was not identified in the genomes of white-eared opossums (Figure 1 and Table S2).

### Characterization of putative satellite DNAs

Satellite DNAs were found to be less frequent than TEs, accounting for 3.64 % of *D. albiventris* and as low as 0.04 % of *D. aurita* genomes (Table 1). Putative satDNA families (hereinafter referred to as satDNA) identified by TAREAN were named by joining the suffix “sat” and the predicted monomer length (Table 2). Only one of these satDNAs (sat206) was detected in all studied species and it was ranked as “high confidence putative satellite” in all species except *D. aurita*. Sat1430 was the other satDNA found in more than a single species (Table 2), being classified as “high confidence putative satellite” for both *D. albiventris* and *D. virginiana*. The remaining satDNAs (sat293, sat345, sat563, sat907, sat2324 and sat4290) were initially detected in individual species, where they were also classified as “low confidence putative satellite” (Table 2). However, similarity-based searches using LAST 1080 indicated contigs with homologous sequences (*i.e.* at least 70 % identity, covering at least 100 bp of alignment length) for every satDNA inferred by TAREAN. Consequently, these contigs were also included in the characterization of the aforementioned satDNA families.

**Table 2 -:** Putative satDNAs identified by the TAREAN pipeline. Values in bold type represent the average probability calculated from the satellite probability inferred for each listed species. Otherwise, the value is the one informed by TAREAN.

Satellite name	Monomer length (bp)	Species	Satellite probability^a^
**sat206**	206	all seven species	0.817
sat293	293	*D. marsupialis*	0.006
sat345	345	*D. virginiana*	0.399
sat563	563	*D. virginiana*	0.670
sat907	907	*D. virginiana*	0.041
**sat1430**	1420	*D. albiventris, D. virginiana*	0.975
sat2320	2320	*D. imperfecta*	0.080
sat4290	4290	*D. marsupialis*	0.093

^a^ “Empirical probability estimate that cluster sequences are derived from satellite repeat”.

This material is available as part of the online article from http://www.scielo.br/gmb

Dot plot graphics demonstrated the existence of high similarity between segments of sat206, sat345 and sat563 and between sat903, at2324 and sat4290 (Figures S1-S5 and S8-S11). In fact, further comparisons showed that sequences of sat206, sat345 and sat563 (Figures S6-S9) shared the same contigs, suggesting that they belong to the same family, but with longer repetition units due to the duplication of internal segments (Figures S8 and S9). A similar result was observed for the pair sat2324 and sat4290. Further analyses were based on comparisons against the shortest monomer of each family, since satDNAs with longer monomers were less frequently represented as complete sequences.

After screening all Repbase database with CENSOR, we found that sat293 was very similar (similarity ≥ 0.90) throughout its length to LINE L1 elements from other marsupial species. This result together with the fact that sat293 was classified as a “low confidence” putative satellite suggest that this family may not be a satDNA, but a segment from an abundant TE. Accordingly, we did not include sat293 in subsequent analyses. Regarding sat206, no significant similarity with TEs was found. Short and low-complexity segments from all the remaining satDNAs presented some degree of similarity with sequences of different repetitive DNAs from unrelated organisms such as invertebrates and plants, therefore implying no significant relationship between satDNA sequences and any known TE.

**Figure 2 -:**
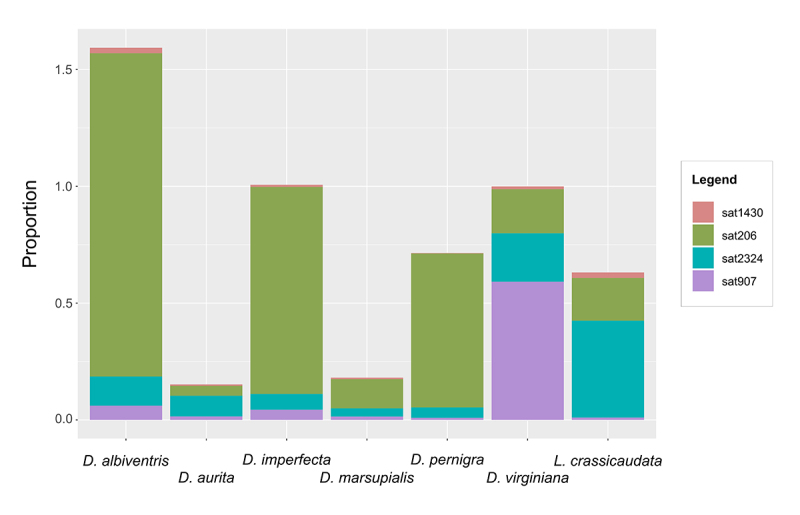
Stacked bar-plot depicting the genome proportion of the satellite DNAs identified by the TAREAN pipeline for *Didelphis* species and *Lutreolina crassicaudata*.

The grouping of sequence contigs based on LAST results allowed us to perform satDNA comparative analyses among species. The first of these comparisons concerns a more comprehensive estimate of the genome abundance for each satDNA. Combined genome proportion of the putative satellite families ranged from 0.152 % in *D. aurita* to 1.593 % in *D. albiventris* (Figure 2 and Table S3). Individually, Sat206 was the most frequent satDNA in four of the studied species: *D. albiventris*, *D. imperfecta*, *D. marsupialis*, and *D. pernigra*. Sat2324 was the most abundant one in *D. aurita* and *L. crassicaudata* (Figure 2 and Table S3). Sat1430, on the other hand, was the least frequent satDNA in all species except for *L. crassicaudata*.

A summary of the main characteristics of all the putative satDNAs is presented in Table 3. However, not all attributes could be calculated for every species or satellite, as was the case of monomer size variation that could only be assessed for sat206, since it was the only satDNA represented by complete monomers sequences in all species. Regarding the other satDNAs, we could not obtain their complete monomer sequences for most species, particularly the longer satDNAs, such as sat1430 and sat2324, in which assembled contigs represented different regions of the satDNA in different species. Considering this result, we prioritized the comparative aspect of the study, hence for each satDNA we tried to select the larger segment yielding the most comprehensive alignment (i.e. we tried to avoid alignments excluding one or more species).

**Table 3 -:** Characterization of putative satDNA families according to the length of the monomer segment analyzed for *Didelphis* species and *Lutreolina crassicaudata*.

SatDNA	Length	%A+T	mean instraspecific distance^a^	max (pairwise distance)^b^	min (pairwise distance)	CENSOR^c^
**sat206**						
*L. crassicaudata*	207	61.70	0.030	0.051	0.000	-
*D. virginiana*	198	57.60	0.053	0.075	0.034	-
*D. aurita*	206	63.10	0.000	-	-	-
*D. marsupialis*	206	64.10	0.008	0.015	0.000	-
*D. albiventris*	196	59.70	0.006	0.010	0.000	-
*D. imperfecta*	196	60.20	0.005	0.010	0.000	-
*D. pernigra*	196	59.20	0.002	0.005	0.000	-
average (satellite)	200.714	60.800	0.015	0.028	0.006	
**sat907**						
*L. crassicaudata*	607	57.30	0.192	0.192	0.192	+
*D. virginiana*	625	60.60	0.144	0.211	0.106	+
*D. aurita*	620	60.20	0.159	0.171	0.150	+
*D. marsupialis*	624	60.70	0.168	0.168	0.168	+
*D. albiventris*	621	59.60	0.160	0.160	0.160	+
*D. imperfecta*	624	61.00	-	-	-	+
*D. pernigra*	625	61.20	0.165	0.201	0.131	+
average (satellite)	620.857	60.086	0.165	0.184	0.151	
**sat1430**						
*L. crassicaudata*	970	69.40	0.001	-	-	+
*D. virginiana*	970	69.60	0.001	-	-	+
*D. aurita*	966	68.60	-	-	-	+
*D. marsupialis*	965	68.30	-	-	-	+
*D. albiventris*	968	68.80	0.005	0.008	0.000	+
*D. imperfecta*	967	68.40	-	-	-	+
*D. pernigra*	667	68.50	-	-	-	+
average (satellite)	924.714	68.800	0.002	0.008	0.000	
**sat2324**						
L. crassicaudata	992	58.80	0.157	0.211	0.100	+
*D. virginiana*	1001	60.90	0.142	0.159	0.122	+
*D. aurita*	998	60.00	0.215	-	-	+
*D. marsupialis*	998	59.70	0.205	0.232	0.152	+
*D. albiventris*	996	59.00	0.175	0.199	0.131	+
*D. imperfecta*	989	59.80	0.148	0.209	0.070	+
*D. pernigra*	987	60.60	0.117	0.205	-	+
average (satellite)	994.429	59.829	0.166	0.203	0.115	

^a^ mean intraspecific Kimura-2-parameter distance. ^b^ maximum and minimum pairwise distance per species. ^c^ “+” on the last column indicate a positive match with a repetitive DNA from Repbase (Bao et al., 2015).

The difference in monomer length of sat206 is mainly explained by the presence of an internal segment (possibly an indel) of 10 bp long in *D. aurita*, *D. marsupialis* and *L. crassicaudata*. This is not the case of the other satDNAs, whose size differences are mainly caused by sequences lacking external segments of varied length (probably incomplete sequences).

All satDNAs presented a slight bias towards an AT-rich content (Table 3), with an average A+T proportion ranging from 59.83 % (sat2324) to 68.8 % (sat1430). Shorter satDNAs, such as sat206, and larger ones, such as sat2324, presented similar A+T content, 60.80 % and 59.83 % respectively. In most cases, A+T content did not seem corelated to phylogenetic relatedness, as even distantly related species showed similar A+T proportion (e.g. *D. aurita* and *D. pernigra* had the same 61.2 % A+T content).

The average intraspecific species sequence variability ranged from 4.2 % (sat206) to 16.6 % (sat2324) (Table 3). We could not assess the sat1430 variability since this family was represented by a single sequence in most species.

**Figure 3 -:**
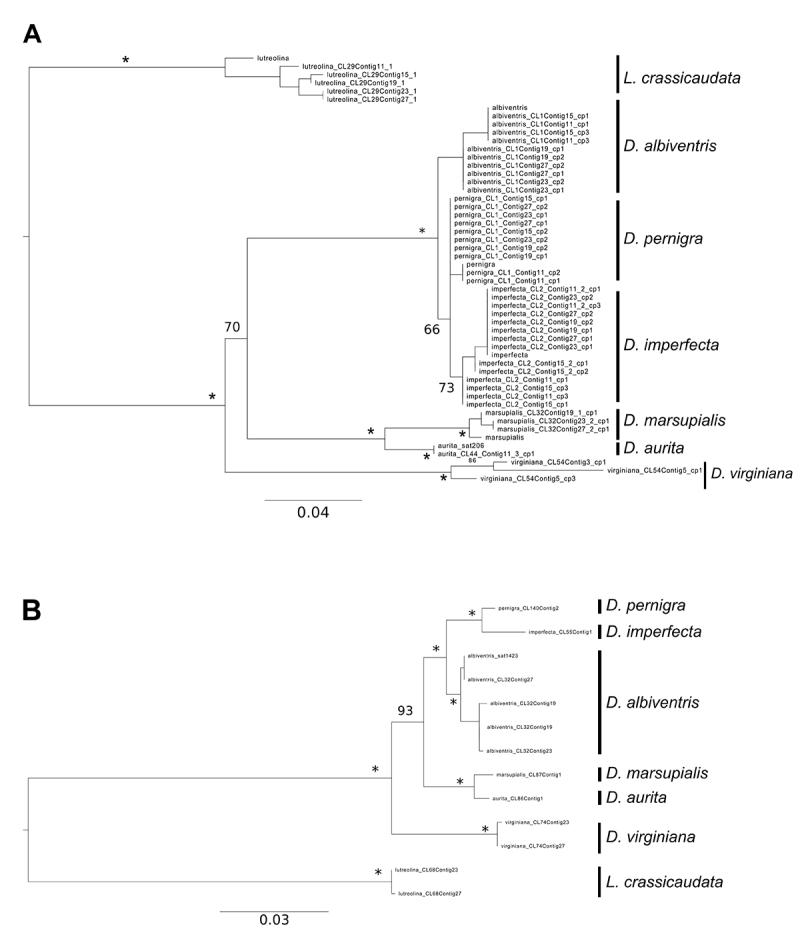
Maximum likelihood trees inferred from putative satellite DNA sequences from (A) sat206 and (B) sat1430. “*” indicates bootstrap values ≥ 95.

Maximum likelihood phylogenetic trees based on satDNA sequences returned mixed results. The two tree topologies inferred by sat206 and by a segment >900 bp from sat1430 (Figure 3) are concordant with the topologies presented in previous studies (Amador and Giannini, 2016; Dias and Perini, 2018; Dias *et al*., 2020), in which *D. virginiana* is recovered as the sister taxon to the remaining *Didelphis* species and both black-eared and white-eared opossums species groups are recovered as monophyletic. Although the phylogenetic relationships among species are not well supported in the analysis based on sat206, most of them showed elevated support (bootstrap ≥ 95) on the tree inferred from sat1430 (Figure 3) and, in both cases, the relationships among white-eared opossums are in agreement with the results of Dias *et al*. (2020), presenting *D. albiventris* as the sister taxon to a clade comprising *D. imperfecta* and *D. pernigra*. Trees inferred from sat907 and sat2324 resulted in unresolved topologies (Figures S12 and S13), where sequences of the same species appeared scattered throughout the tree intermingled with sequences from other species.

## Discussion

The relative genome proportion of TEs observed for the species studied herein are in agreement with what has been reported for the short-tailed mouse opossum (*Monodelphis domestica*), the closest relative marsupial whose genome has been completely sequenced (Mikkelsen et al., 2007). As is the case for other mammals (Deininger et al., 2003), retrotransposons are the most abundant TE in the genomes of these species, and LINEs are the most frequent group of interspersed repeats, a trait that is also shared with other marsupials, such as the tammar wallaby (*Macropus eugenii*) and the Tasmanian devil (*Sarcophilus harrisii*) (Renfree *et al*., 2011; Gallus et al., 2015). Endogenous retroviruses were the second most frequent TE family both in the *Didelphis* species studied and in the *M. domestica* genomes. SINEs, however, which appear to be as frequent as endogenous retroviruses in the *M. domestica* genome (Mikkelsen *et al*., 2007) and are far more abundant than endogenous retroviruses in *M. eugenii* (Graves and Renfree, 2013), are the third most frequent TE in all *Didelphis* species and, unlike LINEs and LTRs, their proportions are not consistently smaller for the white-eared opossums. Most of the contigs were related to two SINE families: SINE_1 MD, which has been previously detected in the genomes of American marsupials (*D. virginiana* and *M.* domestica) and MAR1, which was detected in Australian and American marsupials (Gilbert and Labuda, 1999; Munemasa et al., 2008). Sequences similar to other widespread SINEs, such as Ther-1 and Ther-2 (distributed in therian genomes), were also identified.

Although the overall proportion of TEs and the relative abundance of the three most frequent TE families are very similar among *Didelphis* species, we noted that some patterns in TE count and composition may be specific to the species of *D. albiventris* group, which consistently present a less diverse TE composition and a smaller proportion of LINEs and LTRs in their genomes than the other species analyzed in the present study.

In this study, we also identified and characterized four new putative satDNAs present in all species of *Didelphis* and *L. crassicaudata.* Indeed, other satDNA families were identified by TAREAN, but were not characterized as they were very similar to other satDNAs with higher satellite probabilities. This is the case of sat563, whose monomer consensus sequence seems to contain three copies of varying length of the sat206 monomer consensus (Figures S2 and S9). This relationship was further confirmed by LAST results that showed that these satDNAs referred to the same contigs. Nonetheless, when inspecting longer contigs bearing multiple repeat units, we observed that while white-eared opossums sequences comprised identical copies of the monomer, the other species’ sequences held different copies of it. This pattern suggests that sat206 may exist as higher-order repeat (HOR - when the repeat unit is composed of multiple variants of the monomer) structure in the remaining species. This, in turn, may indicate a recent amplification of the monomer in the black-eared opossums, *D. virginiana* and *L. crassicaudata*, a hypothesis that is corroborated by the small fraction of sat206 in these species when compared to the white-eared opossums, in which sat206 is more abundant and, invariably, the most frequent satDNA.

Yonenaga-Yassuda et al. (1982) compared the C-banding patterns of *D. albiventris, D. marsupialis* and *L. crassicaudata* chromosomes and showed that *D. albiventris* presented centromeric heterochromatin in all autosomes and in the X chromosome, while in *D. marsupialis* and *L. crassicaudata* heterochromatin was only evident in the sex chromosomes. Similarly, our results indicate that the abundance of satDNA in *D. albiventris* is considerably higher than in both *D. marsupialis* and *L. crassicaudata*.

Sequence divergence among copies of a monomer of satDNA is expected to be low, due to concerted evolution leading to the homogenization of the repeats (Plohl and Meštrović, 2012). However, we observed that sequence divergence among satDNA copies within species exceeded 20 % in some instances (Table 3). Garrido-Ramos (2017) enumerates factors affecting concerted evolution that could account for high levels of sequence divergence among copies of satDNAs, such as: little time elapsed since the divergence of compared species; chromosomal location, which can affect recombination rates; and reticulated evolution due to gene flow among taxa. Nevertheless, determining the actual mechanism underlying the observed levels of intraspecific sequence divergence would require further experimental approaches.

Despite corroborating the monophyly of most species and species groups of *Didelphis*, phylogenetic trees based on satDNA sequences could not resolve the relationships in different levels, particularly the relationships among white-eared opossums. The fast evolving nature of satDNAs (Kuhn et al., 2008; Garrido-Ramos, 2017) may contribute to a rapid loss of phylogenetic information, or maybe the number of sequences from individual satDNAs retrieved during our study was not enough to capture the variability exhibited by some diverse satDNA families.

Our study is one of the first attempts to bring on an *in silico* identification and characterization of the most abundant families of repetitive DNAs of *L. crassicaudata* and all species of the genus *Didelphis* providing insights into the participation of repetitive DNAs in the genome landscape of marsupial species whose genomes have not been completely sequenced yet. Our results serve as a starting point for experimental cytogenetic analyses looking for an in-depth understanding of the Didelphini chromosomal evolution.

## Supplementary material

The following online material is available for this article:

Table S1 **-** General sequencing information.

Table S2 - Genome proportion of families of transposable elements with abundance of at least 0.01 % in *Didelphis* species and *Lutreolina crassicaudata*.

Table S3 - Genome proportion of families of putative satellite DNA in *Didelphis* species and *Lutreolina crassicaudata*.

Figure S1 - Dot-plot demonstrating that sat206 is embedded within sat345.

Figure S2 - Dot-plot demonstrating that sat206 is embedded within sat563.

Figure S3 - Dot-plot demonstrating that sat345 is embedded within sat563.

Figure S4 - Dot-plot demonstrating that a segment of sat907 is embedded within sat2324.

Figure S5 - Dot-plot demonstrating that sat2324 is embedded within sat4291.

Figure S6 - Self-similarity dot-plot of sat345.

Figure S7 - Self-similarity dot-plot of sat563 demonstrating it may be composed of smaller segments of repetitive DNA.

Figure S8 - Schematic diagram depicting the identity relationship between SAT206 and SAT345.

Figure S9 - Schematic diagram depicting the identity relationship between SAT206 and SAT563.

Figure S10 - Schematic diagram depicting the identity relationship between SAT907 and SAT2324.

Figure S11 - Schematic diagram depicting the identity relationship between SAT2324 and SAT4290.

Figure S12 **-** Maximum likelihood phylogeny inferred form sequences of the putative satellite DNA sat907.

Figure S13 - Maximum likelihood phylogeny inferred form sequences of the putative satellite DNA sat2324.
