# Supplementary material for: Identification and characterization of repetitive DNA in the genus Didelphis Linnaeus, 1758 (Didelphimorphia, Didelphidae) and the use of satellite DNAs as phylogenetic markers
Source: Genet Mol Biol. 2021 Apr 16;44(2):e20200384. doi: 10.1590/1678-4685-GMB-2020-0384 (PMC8056902; doi:10.1590/1678-4685-GMB-2020-0384)
Supplement: Table S1 - [file 1415-4757-GMB-44-2-e20200384-s1.pdf]

*Genetics and Molecular Biology*

https://doi.org/10.1590/1678-4685-GMB-2020-0384

# "Supplementary Material to "Identification and characterization of repetitive DNA in the genus Didelphis Linnaeus, 1758 (Didelphimorphia, Didelphidae) and the use of satellite DNAs as phylogenetic markers."

**Table S1** - General sequencing information. Bold values (genome size column) were converted from picograms to base pairs using the equation presented in Dolezel et al. (2003).

| species | raw reads | clean reads | genome size | coverage |
|---|---|---|---|---|
| *Didelphis albiventris* | 6.31E+07 | 5.84E+07 | - | - |
| *Didelphis aurita* | 2.97E+07 | 2.75E+07 | **4205400000** | 0.979[a] |
| *Didelphis imperfecta* | 4.17E+07 | 3.84E+07 | - | - |
| *Didelphis marsupialis* | 4.08E+07 | 3.77E+07 | 3.14E+09 | 1.803[b] |
| *Didelphis pernigra* | 6.22E+07 | 5.74E+07 | - | - |
| *Didelphis virginiana* | 4.22E+07 | 3.83E+07 | **4058700000** | 1.415[c] |
| *Lutreolina crassicaudata* | 3.84E+07 | 3.55E+07 | **4107600000** | 1.311[a] |

[a]Garagna & Formenti (1981), [b]Redi et al. (2005), [c]Tiersch et al. (1989)

**References**

Dolezel, J., Bartos, J., Voglmayr, H., & Greilhuber, J. (2003). Nuclear DNA content and genome size of trout and human. Cytometry, 51A(2), 127–128. https://doi.org/10.1002/cyto.a.10013

Garagna, S., & Formenti, D. (1981). DNA content variability in several species of Australian and South American marsupials. Bolletino Di Zoologia, 48(3–4), 255–261. https://doi.org/10.1080/11250008109439342

Redi, C. A., Zacharias, H., Merani, S., Oliveira-Miranda, M., Aguilera, M., Zuccotti, M., Garagna, S., & Capanna, E. (2005). Genome sizes in Afrotheria, Xenarthra, Euarchontoglires, and Laurasiatheria. Journal of Heredity, 96(5), 485–493. https://doi.org/10.1093/jhered/esi080
