# Supplementary material for: Identification and characterization of repetitive DNA in the genus Didelphis Linnaeus, 1758 (Didelphimorphia, Didelphidae) and the use of satellite DNAs as phylogenetic markers
Source: Genet Mol Biol. 2021 Apr 16;44(2):e20200384. doi: 10.1590/1678-4685-GMB-2020-0384 (PMC8056902; doi:10.1590/1678-4685-GMB-2020-0384)
Supplement: Table S2 - [file 1415-4757-GMB-44-2-e20200384-s2.pdf]

Genetics and Molecular Biology

https://doi.org/10.1590/1678-4685-GMB-2020-0384

"Supplementary Material to "Identification and characterization of repetitive DNA in the genus Didelphis Linnaeus, 1758 (Didelphimorphia, Didelphidae) and the use of satellite DNAs as phylogenetic markers."

Table S2 - Genome proportion of families of transposable elements with abundance of at least 0.01% in *Didelphis* species and *L. crassicaudata.*

	Genome proportion (%)						
**repetitive DNA**	***D. albiventris***	***D. aurita***	***D. imperfecta***	***L. crassicaudata***	***D. marsupialis***	***D. pernigra***	***D. virginiana***
**Retrotransposons**							
CR1	-	-	-	0.0828	0.0115	-	0.0448
L1	11.1421	12.2224	11.7362	14.7905	12.7786	11.5197	12.7774
RTE	-	0.0122	-	0.0632	0.0180	-	0.0192
SINE	-	-	-	0.0493	0.0220	-	-
LTR							
Endogenous_Retrovirus	1.3136	1.7112	1.4386	1.3728	1.8350	1.3440	1.8862
ERV1	1.1010	1.4512	1.0145	0.9657	1.3810	1.0883	1.6851

	Genome proportion (%)						
**repetitive DNA**	***D. albiventris***	***D. aurita***	***D. imperfecta***	***L. crassicaudata***	***D. marsupialis***	***D. pernigra***	***D. virginiana***
ERV2	0.0279	0.0637	0.0372	0.0660	0.0486	0.0368	0.0805
Gypsy	-	-	-	-	0.0149	-	-
**DNA transposons**							
DNANA5_MD	-	-	-	0.0394	-	-	0.0156
Mariner/Tc1	-	0.0134	-	0.0158	0.0168	-	0.0135
hAT	-	0.0234	-	0.0342	0.0220	0.0149	0.0193
**Total**	**13.5845**	**15.4975**	**14.2265**	**17.4795**	**16.1485**	**14.0038**	**16.5414**
