# Supplementary material for: Identification and characterization of repetitive DNA in the genus Didelphis Linnaeus, 1758 (Didelphimorphia, Didelphidae) and the use of satellite DNAs as phylogenetic markers
Source: Genet Mol Biol. 2021 Apr 16;44(2):e20200384. doi: 10.1590/1678-4685-GMB-2020-0384 (PMC8056902; doi:10.1590/1678-4685-GMB-2020-0384)
Supplement: Table S3 - [file 1415-4757-GMB-44-2-e20200384-s3.pdf]

Genetics and Molecular Biology

https://doi.org/10.1590/1678-4685-GMB-2020-0384

“Supplementary Material to “Identification and characterization of repetitive DNA in the genus Didelphis Linnaeus, 1758 (Didelphimorphia, Didelphidae) and the use of satellite DNAs as phylogenetic markers.”

Table S3 - Genome proportion of families of putative satellite DNA in *Didelphis* species and *L. crassicaudata.*

	Genome proportion (%)						
**satDNA**	***D. albiventris***	***D. aurita***	***D. imperfecta***	***D. marsupialis***	***D. pernigra***	***D. virginiana***	***L. crassicaudata***
sat1430	0.0237	0.0062	0.0090	0.0067	0.0018	0.0119	0.0235
sat206	1.3840*	0.0427	0.8860*	0.1247	0.6593*	0.1886	0.1833
sat2324	0.1247	0.0881	0.0677	0.0350	0.0447	0.2074	0.4145
sat907	0.0612	0.0152	0.0438	0.0148	0.0087	0.5917	0.0103
**Total**	**1.5935**	**0.1522**	**1.0064**	**0.1812**	**0.7144**	**0.9996**	**0.6315**

* genome proportion calculation took into account only the number of reads in the respective satellite cluster (but not the supercluster), which led to a reduced overall satellite DNA proportion when compared to table 1.
