# Supplementary material for: Identification and characterization of repetitive DNA in the genus Didelphis Linnaeus, 1758 (Didelphimorphia, Didelphidae) and the use of satellite DNAs as phylogenetic markers
Source: Genet Mol Biol. 2021 Apr 16;44(2):e20200384. doi: 10.1590/1678-4685-GMB-2020-0384 (PMC8056902; doi:10.1590/1678-4685-GMB-2020-0384)

Genetics and Molecular Biology

https://doi.org/10.1590/1678-4685-GMB-2020-0384

"Supplementary Material to "Identification and characterization of repetitive DNA in the genus Didelphis Linnaeus, 1758 (Didelphimorphia, Didelphidae) and the use of satellite DNAs as phylogenetic markers."

Figure S5 - Dot-plot demonstrating that sat2324 is embedded within sat4291.

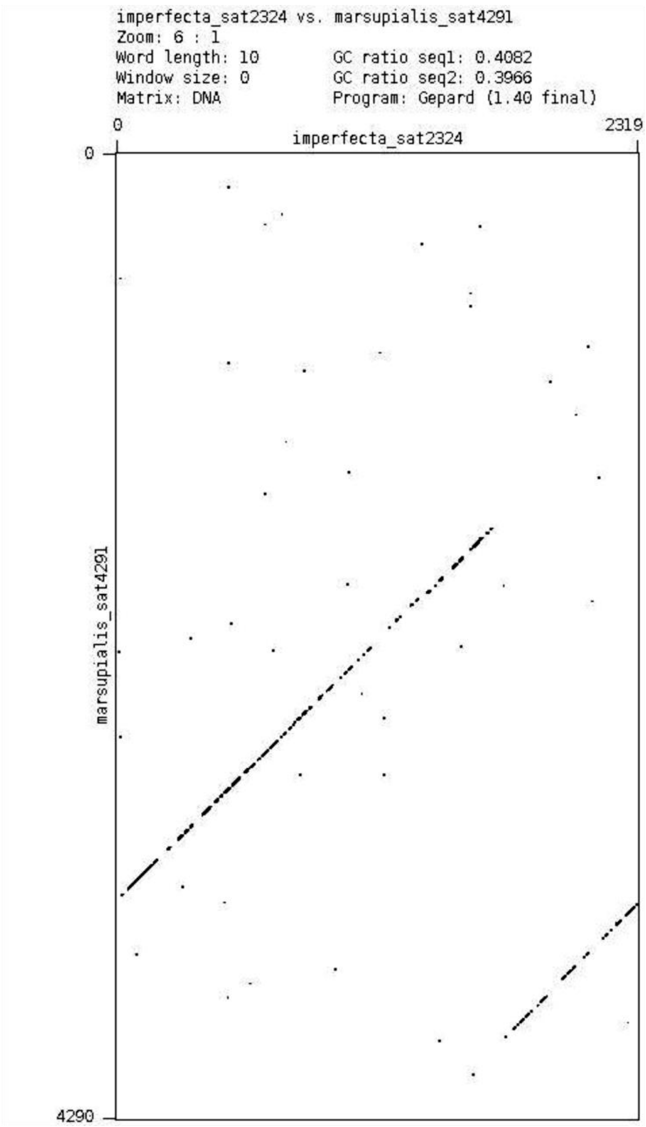

Supplement: Figure S5 - [file 1415-4757-GMB-44-2-e20200384-s8.pdf]
